# Genome-Wide Identification, Characterization and Evolutionary Analysis of Long Intergenic Noncoding RNAs in Cucumber

**DOI:** 10.1371/journal.pone.0121800

**Published:** 2015-03-23

**Authors:** Zhiqiang Hao, Chunyan Fan, Tian Cheng, Ya Su, Qiang Wei, Guanglin Li

**Affiliations:** 1 College of Life Sciences, Shaanxi Normal University, Xi’an, China; 2 Co-Innovation Center for Qinba Regions’ Sustainable Development, College of Life Sciences, Shaanxi Normal University, Xi’an, China; Kunming University of Science and Technology, CHINA

## Abstract

Long intergenic noncoding RNAs (lincRNAs) are intergenic transcripts with a length of at least 200 nt that lack coding potential. Emerging evidence suggests that lincRNAs from animals participate in many fundamental biological processes. However, the systemic identification of lincRNAs has been undertaken in only a few plants. We chose to use cucumber (*Cucumis sativus*) as a model to analyze lincRNAs due to its importance as a model plant for studying sex differentiation and fruit development and the rich genomic and transcriptome data available. The application of a bioinformatics pipeline to multiple types of gene expression data resulted in the identification and characterization of 3,274 lincRNAs. Next, 10 lincRNAs targeted by 17 miRNAs were also explored. Based on co-expression analysis between lincRNAs and mRNAs, 94 lincRNAs were annotated, which may be involved in response to stimuli, multi-organism processes, reproduction, reproductive processes, and growth. Finally, examination of the evolution of lincRNAs showed that most lincRNAs are under purifying selection, while 16 lincRNAs are under natural selection. Our results provide a rich resource for further validation of cucumber lincRNAs and their function. The identification of lincRNAs targeted by miRNAs offers new clues for investigations into the role of lincRNAs in regulating gene expression. Finally, evaluation of the lincRNAs suggested that some lincRNAs are under positive and balancing selection.

## Introduction

The majority of the genome can be transcribed into RNA, but only a small fraction of these transcripts can be translated into proteins [1–3]. In addition to protein-coding RNA, tRNA, rRNA, and many small noncoding RNAs have been discovered, including miRNA, siRNA and piRNA [4, 5]. Long intergenic noncoding RNAs (lincRNAs) are a newly described type of noncoding RNA derived from the intergenic regions of the genome [6–9]. LincRNAs generally exhibit a length of at least 200 nt and lack coding potential. They can regulate gene expression at the transcriptional and post-transcriptional levels by acting as signals, decoys, guides, and scaffolds [10]. Emerging evidence suggests that lincRNAs from animals participate in many biological processes, including cell-cycle regulation, immune surveillance, and embryonic stem cell pluripotency [11–14].

Due to the development of genomic sequencing techniques, genome-wide identification of lincRNAs can be achieved via cDNA/EST, Chip-seq, tilling array and RNA-seq data analyses. The identification of lincRNAs has been widely reported in higher eukaryotes, including humans, chickens, pigs, and flies [11, 15–18]. However, the systematic identification of lincRNAs in plants has received less attention. In *Arabidopsis thaliana*, 6,480 lincRNAs were identified using custom arrays and RNA sequencing [19]. In *Setaria italic*, 584 long noncoding RNAs (lncRNAs), 494 of which are lincRNAs, were identified from a set of full-length cDNAs [20]. In *Zea mays*, 2,492 lncRNAs, 54% of which are lincRNAs, were identified using a pipeline combined with CPC [21]. Another study focused on *Zea mays* identified 1,704 HC-lncRNAs from a comprehensive set of transcripts, 93% of which are lincRNAs [22]. In *Populus trichocarpa*, 2,542 lincRNAs were identified [23]. Although many lincRNAs have been identified, their function is largely unknown.

Compared with protein-coding genes, the orthologs of lincRNAs are less conserved in other species and exhibit high rates of sequence evolution. This makes the identification of lincRNAs and prediction of their function based only on sequence conservation infeasible. To overcome this difficulty, it is urgent to expand investigations of the function and evolution of plant lincRNAs to include more plant species. Cucumber (*Cucumis sativus*) is an important vegetable and serves as a model plant for the study of sex determination and fruit development [24, 25]. Noncoding RNAs from cucumbers play important roles in regulating gene expression. For example, *CR20* is a cytokinin-repressed noncoding gene [26], and *CsM10* is a noncoding gene expressed preferentially under male expression conditions [27]. Because of the importance of cucumbers in both daily life and plant research, many cucumber genomes and transcriptomes have been sequenced. The resulting data, which are publically accessible, enable the potential identification, characterization and evolutionary analysis of cucumber lincRNAs on a genome-wide scale.

In this study, lincRNAs are first identified and characterized on a genomic scale by applying a pipeline to cucumber transcriptome data. Next, the regulatory relationships between miRNAs and lincRNAs are explored. The expression and function of lincRNAs are then investigated based on lincRNA-mRNA co-expression networks. Finally, the evolution of lincRNAs is analyzed. Our results provide a rich resource for studies investigating the functions of lincRNAs in cucumbers and offer insights into the roles of lincRNAs in plants.

## Results

### Genome-wide identification of lincRNAs in cucumbers

**Fig 1:**
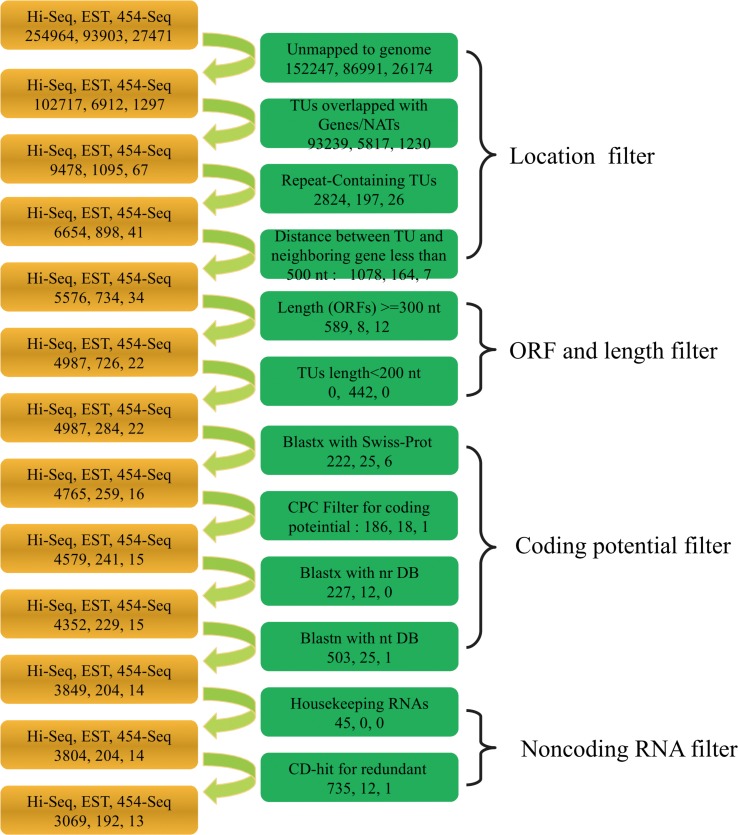
Pipeline for lincRNA identification. The left frames contain the number of transcripts that passed the previous filter. The right frames show the process of filtering and the screened transcript numbers. Three sources were used to identify lincRNAs: hi-seq, EST, and 454-seq data. Hi-Seq: Illumina high-throughput RNA Sequencing; EST: expressed sequence tags; 454-Seq: 454-pyrosequencing; CPC: coding potential calculator; TU: transcript unit.

To comprehensively identify lincRNAs, cucumber transcriptome data (S1 Table) from expressed sequence tag (EST), Illumina high-throughput sequencing (Hi-seq) and 454 pyrosequencing (454-seq) were first assembled and integrated and subsequently subjected to a modified pipeline (Fig. 1) [19, 21, 22]. In total, 110,926 transcripts (6,912 EST, 102,717 assembled Hi-seq, and 1,297 assembled 454-seq transcripts) were mapped to the genome without mismatches. Next, two classical filters were used: one to eliminate transcripts that overlap with repeat elements and annotated protein-coding genes, and a second to filter out both transcripts with long ORFs (> = 300 nt) and short lengths (< 200 nt). BLAST and the coding potential calculator (CPC) were employed to remove sequences with coding potential [28]. Finally, 4,067 transcripts were identified as putative intergenic noncoding RNAs.

Because the remaining putative long intergenic noncoding RNAs may contain housekeeping ncRNAs, such as tRNAs, rRNAs, snoRNAs, and snRNAs, we subjected these putative intergenic noncoding RNAs to BLAST searches against the Rfam database with a threshold E-value of ≤ 1e-10 and identified 4,022 lincRNAs [29]. After removing redundant lincRNAs from the merged datasets, 3,274 lincRNAs that passed all pipeline criteria were regarded as lincRNA candidates. Information on genomic positions is provided in S2 Table. The 3,274 lincRNAs mapped to 3,298 positions in the cucumber genome, with 7 lincRNAs showing multiple genomic positions. From these data, we can infer that lincRNAs tend to be distributed unevenly across seven chromosomes (chi-square goodness of fit test, *p*-value = 0.0007849). Chromosome three contained the largest number of lincRNAs, while chromosome seven presented the fewest (S1 Fig.).

### Characteristics and conservation of cucumber lincRNAs

**Fig 2:**
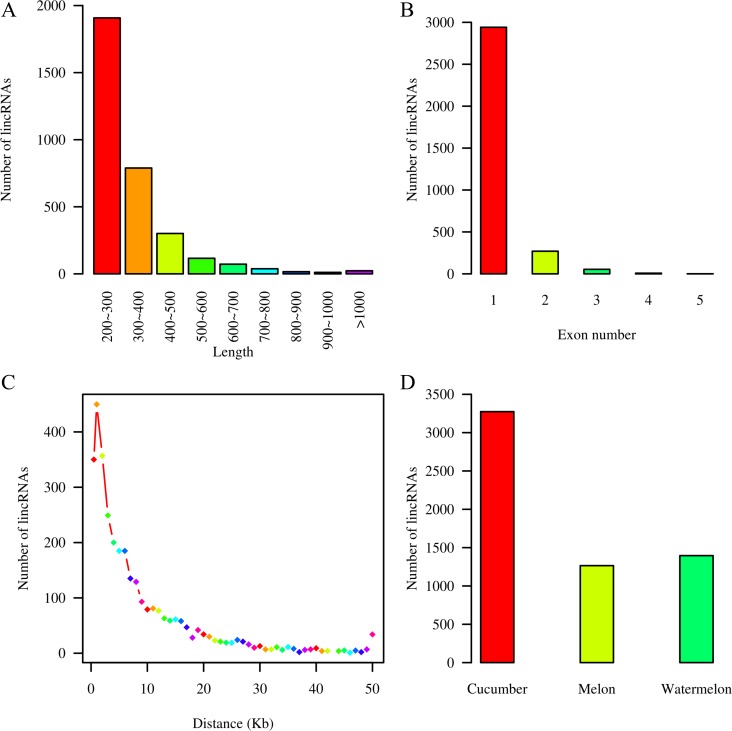
Characteristics of lincRNAs. (A) Length distribution of 3,274 lincRNAs. The X-axis represents the length of lincRNAs. The Y-axis represents the number of lincRNAs with specific lengths. (B) Distribution of exon numbers. The X-axis displays the exon numbers; the Y-axis shows the number of lincRNAs corresponding to specific exon numbers. (C) The nearest distance between lincRNAs and their neighboring protein-coding genes. (D) The conservation of lincRNAs.

The length of the lincRNAs ranged from 200 to 2,573 nucleotides (nt), the majority of which (58.3%) were approximately 200∼300 nt in length (Fig. 2A). The mean length was 322 nt, which is lower than the values observed for cucumber mRNAs (mean length = 1,433 nt). The short length of the lincRNAs may be explained by the reason that these transcripts are not full-length cDNAs. Despite this, the length of the cucumber lincRNAs is comparable to that of the lincRNAs identified in *Arabidopsis thaliana* [19]. Gene structure analysis showed that the majority (89%) of the lincRNAs contain only a single exon (Fig. 2B). More than half of the lincRNAs (51.3%) exhibit a distance more than 5 Kb from their neighboring protein-coding genes, and only 350 (10.7%) of the lincRNAs overlap the flanking regions (0.5∼1 Kb) of neighboring protein-coding genes. This suggests that most of the lincRNAs are transcribed independently from neighboring protein-coding genes (Fig. 2C).

To investigate lincRNA conservation, 3,274 lincRNA sequences were subjected to BLAST searches against the genome sequences of 10 representative plants (*P*. *patens*, *S*. *moellendorffii*, *P*. *abies*, *O*. *sativa*, *Z*. *mays*, *A*. *thaliana*, *P*. *trichocarpa*, *V*. *vinifera*, watermelon, and melon) with a threshold E-value of ≤ 1e-10. We defined conserved lincRNAs as those with more than 20% of their sequence matched to other genomes. Our results indicate that 42% and 38% of cucumber lincRNAs are conserved compared with watermelon and melon, respectively (Fig. 2D, S3 Table). However, only a few short lincRNA elements of approximately 20∼40 bp were conserved compared with the other eight distantly related species (E-value ≤ 1e-5) (S2 Fig.). These results imply that lincRNAs undergo rapid evolution.

### LincRNA expression patterns in different tissues

**Fig 3:**
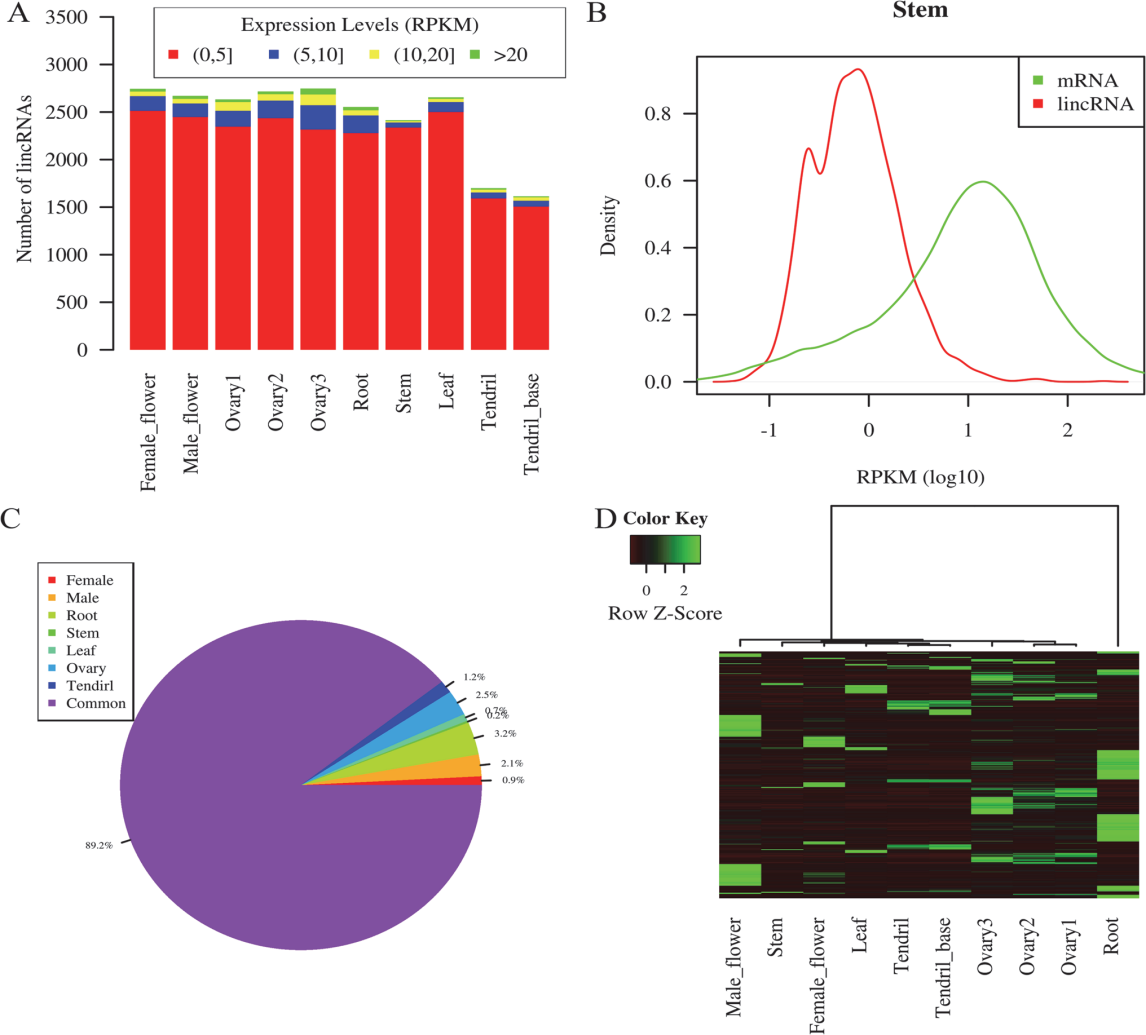
Expression pattern of lincRNAs. (A) The numbers of lincRNAs showing different expression levels in each tissue. (B) Different expression levels of lincRNAs and mRNAs in the stem. (C) Proportion of lincRNAs exhibiting tissue-specific expression in different tissues. (D) Heat map of lincRNAs with tissue-specific expression.

The expression pattern of lincRNAs (RPKM, reads per kilobase per million reads) was explored using RNA-seq data from 10 different tissue types: root, stem, leaf, male flowers, female flowers, ovary, expanded fertilized ovary (7 days after flowering), expanded unfertilized ovary (7 days after flowering), base of the tendril, and tendril. Based on the maximum expression level of each lincRNA in all 10 tissues (exp_*max*_), the expression of the lincRNAs can be divided into four classes (Fig. 3A): (1) low (exp_*max*_ ≤ 5 RPKM); (2) moderate (exp_*max*_ > 5 RPKM and exp_*max*_ ≤ 10 RPKM); (3) high (exp_*max*_ > 10 RPKM and exp_*max*_ ≤ 20 RPKM); and (4) very high (exp_*max*_ > 20 RPKM). While in each tissue, the majority of lincRNAs belong to the low class based on lincRNA expression, some lincRNAs belong to the moderate, high or very high class, indicating that the lincRNAs exhibit a biological purpose, rather than simply representing transcriptional “noise”.

Kernel density estimates (KDE) of gene expression were used to compare the expression levels of lincRNAs and mRNAs. We found that lincRNAs and mRNAs exhibit different density peaks and that the density peaks of mRNAs lag behind those of lincRNAs in each tissue (Fig. 3B, S3A-S3I Figs.). Based on these results, we can infer that lincRNAs display lower expression levels than mRNAs in each tissue (Kolmogorov-Smirnov test, *p* < 2.2×10^–16^), which is consistent with the expression of lincRNAs in *Arabidopsis thaliana* [19].

The tissue-specific expression of lincRNAs was investigated using the tissue-specific index [30]. Overall, the roots displayed the most diverse lincRNA expression levels and presented the largest number of tissue-enriched lincRNAs (Fig. 3C, S4 Table). Approximately 10.8% of all lincRNAs (353 of 3,274) presented enriched expression in a single tissue, and 30% (105 of 353) were highly enriched in the roots. Heat maps for all tissue-enriched lincRNAs showed that not only do the roots display the largest number of tissue-enriched lincRNAs, most of the enriched lincRNAs are in the high expression group (Fig. 3D, S4 Table).

### LincRNAs are potential targets or target mimics of cucumber miRNAs

**Fig 4:**
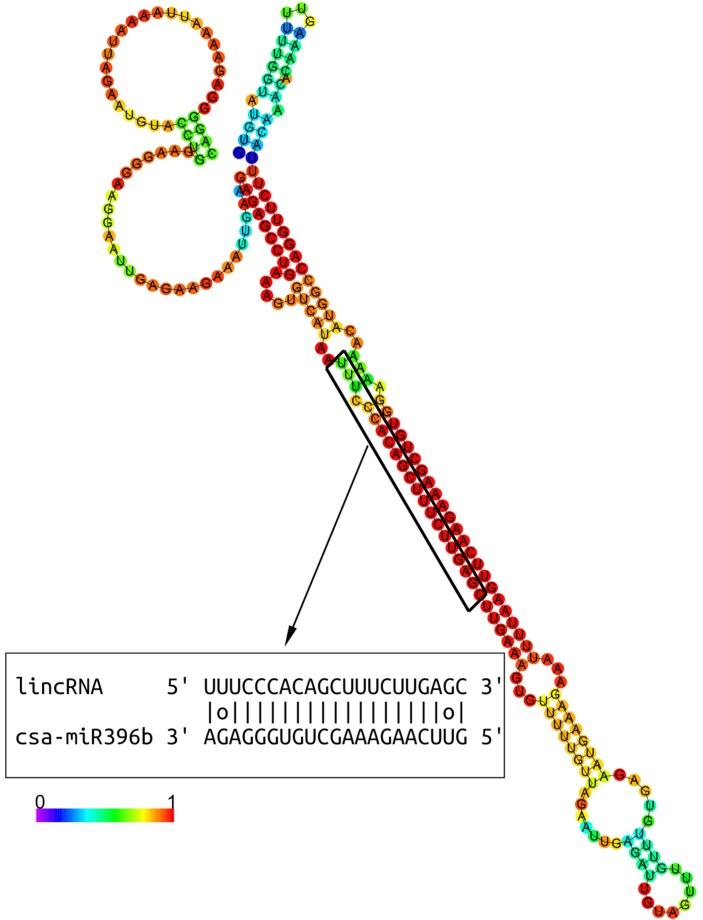
LincRNAs targeted by miRNAs. Secondary structure of one lincRNA (CU1NC272) and the base-pairing relationship between the lincRNA (CU1NC272) and Csa-miRNA396b. The predicted secondary structure was generated using RNAfold (minimum free energy: −63.50).

Studies have shown that lincRNAs may act as targets or target mimics of miRNAs to regulate gene expression. For example, a lincRNA referred to as *IPS1* (*induced by phosphate starvation 1*) acts as a target mimic of miR-399 in *Arabidopsis thaliana* [31]. To investigate the relationship between miRNAs and lincRNAs, we predicted potential miRNA targets or target mimics in lincRNAs using psRobot, a widely employed miRNA target prediction tool, and identified 10 lincRNAs as potential targets or target mimics of 17 miRNAs (Table 1). For example, one of the cucumber lincRNAs (CU1NC272) targeted by csa-miRNA396b is presented (Fig. 4). According to the sequence conservation of these miRNAs, these 17 miRNAs can be divided into 8 families, including miRNA156, miRNA159/miRNA319, miRNA162, miRNA166, miRNA172, miRNA396, miRNA399 and miRNAn2. Given the significant regulatory impact of miRNAs on their target mRNAs, we infer that these lincRNAs function as miRNA targets or target mimics that may be involved in the miRNA-mRNA network.

**Table 1:** LincRNAs targeted by miRNAs.

**miRNA_Term**	**LincRNA_Term**	**Score** ^a^	**Alignment** ^b^
csa-miR156a	CU2NC895	0	miRNA: 1 GCTCACTTCTCTCTCTGTCAGA 22
			||||||||||||||||||||||
			lincRNA: 304 CGAGTGAAGAGAGAGACAGTCT 283
csa-miR156b	CU2NC895	1	miRNA: 1 GCTCACTTCTCTTTCTGTCAG-T 22
			||||||||||||:|||||||| |
			lincRNA: 304 CGAGTGAAGAGAGAGACAGTCTA 282
csa-miR156d	CU7NC3095	2.5	miRNA: 1 TGCCAGAAGAGAGTGAGCAC 20
			|:|||||||||||: ||||
			lincRNA: 108 ATGGTCTTCTCTCTTTCGTT 89
csa-miR159c	CU5NC2102	2.5	miRNA: 1 TTTGGATTGAAGGGAGCTCT 20
			||||:||||||:||||||
			lincRNA: 228 TCACCTGACTTCCTTCGAGA 209
csa-miR162a	CU6NC2683	2.2	miRNA: 1 GGAGGCAGCGGTTCATCGACC 21
			: | ||||:||||||||||||
			lincRNA: 142 TCGCCGTTGCCAAGTAGCTGT 122
csa-miR166	CU2NC947	0.8	miRNA: 1 TCGGACCAGGCTTCATTC-TCG 21
			|||||||||||||||||| ||:
			lincRNA: 275 AGCCTGGTCCGAAGTAAGGAGT 254
csa-miR172c	CU3NC1224	2.5	miRNA: 1 GAGAATCTTGATGATGCTGCA 21
			|||||||||||| ||| |||
			lincRNA: 246 CTCTTAGAACTAATACTTCGT 226
csa-miR319	CU5NC2102	1.5	miRNA: 1 TTGGACTGAAGGGAGCTCCCT 21
			|||||||||||:||||| ||
			lincRNA: 227 CACCTGACTTCCTTCGAGAGA 207
csa-miR396a	CU1NC272	1	miRNA: 1 CCACAGCTTTCTTGAACTGCA 21
			|||||||||||||||||| |
			lincRNA: 181 GGTGTCGAAAGAACTTGAATT 161
csa-miR396b	CU1NC272	0.8	miRNA: 1 GTTCAAGAAAGCTGTGGGAGA 21
			|:|||||||||||||||||:|
			lincRNA: 102 CGAGTTCTTTCGACACCCTTT 82
csa-miR396c	CU1NC272	1.8	miRNA: 1 GTTCAATAAAGCTGTGGGAAG 21
			|:|||| |||||||||||||:
			lincRNA: 102 CGAGTTCTTTCGACACCCTTT 82
csa-miR396d	CU1NC272	0	miRNA: 1 TTCCACAGCTTTCTTGAACTT 21
			|||||||||||||||||||||
			lincRNA: 183 AAGGTGTCGAAAGAACTTGAA 163
csa-miR399a	CU5NC2035	0	miRNA: 1 AGGGCTTCTCTCCATTGGCAGG 22
			||||||||||||||||||||||
			lincRNA: 818 TCCCGAAGAGAGGTAACCGTCC 797
csa-miR399b	CU6NC2935	2	miRNA: 1 TGCCAAAAGAGACTTGCCC 19
			|||||| |||||||:|| |
			lincRNA: 148 ACGGTTATCTCTGAGCGTG 130
csa-miR399b	CU5NC2035	2	miRNA: 1 TGCCAAAAGAGACTTGCCC 19
			||||||| |||| ||||||
			lincRNA: 771 ACGGTTTCCTCTCAACGGG 753
csa-miR399c	CU5NC2035	0	miRNA: 1 TGCCAAAGGAGAGTTGCCCTT 21
			|||||||||||||||||||||
			lincRNA: 771 ACGGTTTCCTCTCAACGGGAA 751
csa-miR399d	CU5NC2035	2	miRNA: 1 TGCCAAAGGAGATTTGCCCGG 21
			|||||||||||| ||||||
			lincRNA: 771 ACGGTTTCCTCTCAACGGGAA 751
csa-miRn2–3p	CU5NC2296	2.5	miRNA: 1 ATCTAACGATGTAGGAGCAAT 21
			|| |||||||:|||:|||||
			lincRNA: 210 TACATTGCTATATCTTCGTTT 190

^a^ indicates the total score of the alignment.

^b^ indicates the alignment between the miRNA and target (lincRNA).

### Function of cucumber lincRNAs

#### Co-expression of lincRNAs and mRNAs in cucumber

**Fig 5:**
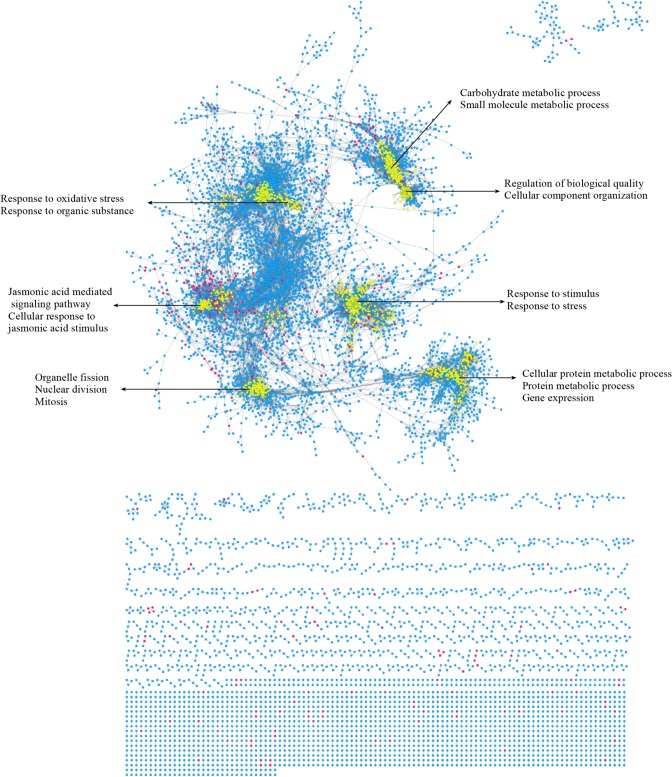
LincRNA-mRNA co-expression network. Nodes with red circles represent lincRNAs, and nodes with blue circles represent mRNAs. The edges represent connected nodes that exhibit a high correlation. Several large modules highlighted in yellow are also shown and annotated according to BP (Biological Processes).

To infer the function of lincRNAs, a co-expression network between lincRNAs and mRNAs was constructed and visualized (see method for details). There were 207,341 relationships included in the network, including 10,794 mRNAs and 440 lincRNAs (Fig. 5). Specifically, 194,290 of the links were between two mRNAs, while 12,522 were between lincRNAs and mRNAs, and 529 were between two lincRNAs. Among all 440 lincRNAs included in the network, 388 exhibited at least one mRNA as a partner, involving 2,347 mRNAs.

#### Function prediction of cucumber lincRNAs based on the co-expression network

Two methods were employed to mine the function of lincRNAs based on the lincRNA-mRNA co-expression network. Using the hub-based method, 126 lincRNAs were identified as hub genes (each hub gene has at least ten coding genes as partners). Under the module-based method, 34 modules involving 135 lincRNAs were identified (each module has at least ten coding genes and one lincRNA).

**Fig 6:**
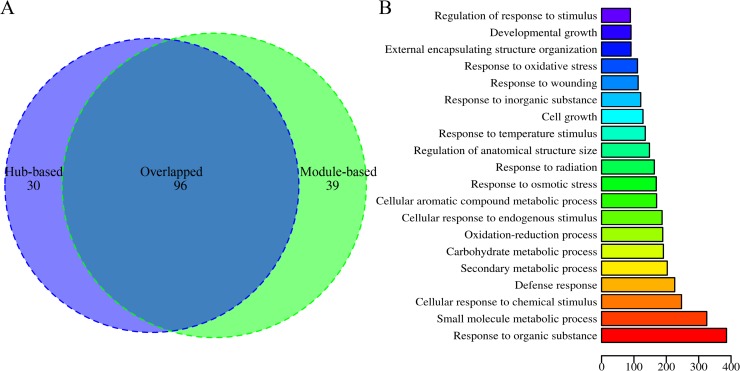
Functions of lincRNAs. (A) A Venn Diagram showing the number of lincRNAs predicted via the hub-based and module-based methods. (B) The main biological processes (BP) of overlapping lincRNAs predicted by two methods. The X-axis indicates the number of enriched mRNAs.

In total, 96 lincRNAs overlapped in the results from the hub-based and module-based methods (Fig. 6A). Furthermore, 94 lincRNAs showed functional annotations with at least one GO term: BP (biological processes, including the response to stimulus, multi-organism processes, reproduction, reproductive processes, growth, and others); MF (molecular functions, including metal ion binding, oxidoreductase activity, transporter activity, hydrolase activity, heme binding, and others); or CC (cell components, including cell parts, membrane parts, the apoplast, and extracellular region parts) (Fig. 6B, S4A and S4B Figs., S5 and S6 Tables).

### Polymorphism and evolution of cucumber lincRNAs

**Fig 7:**
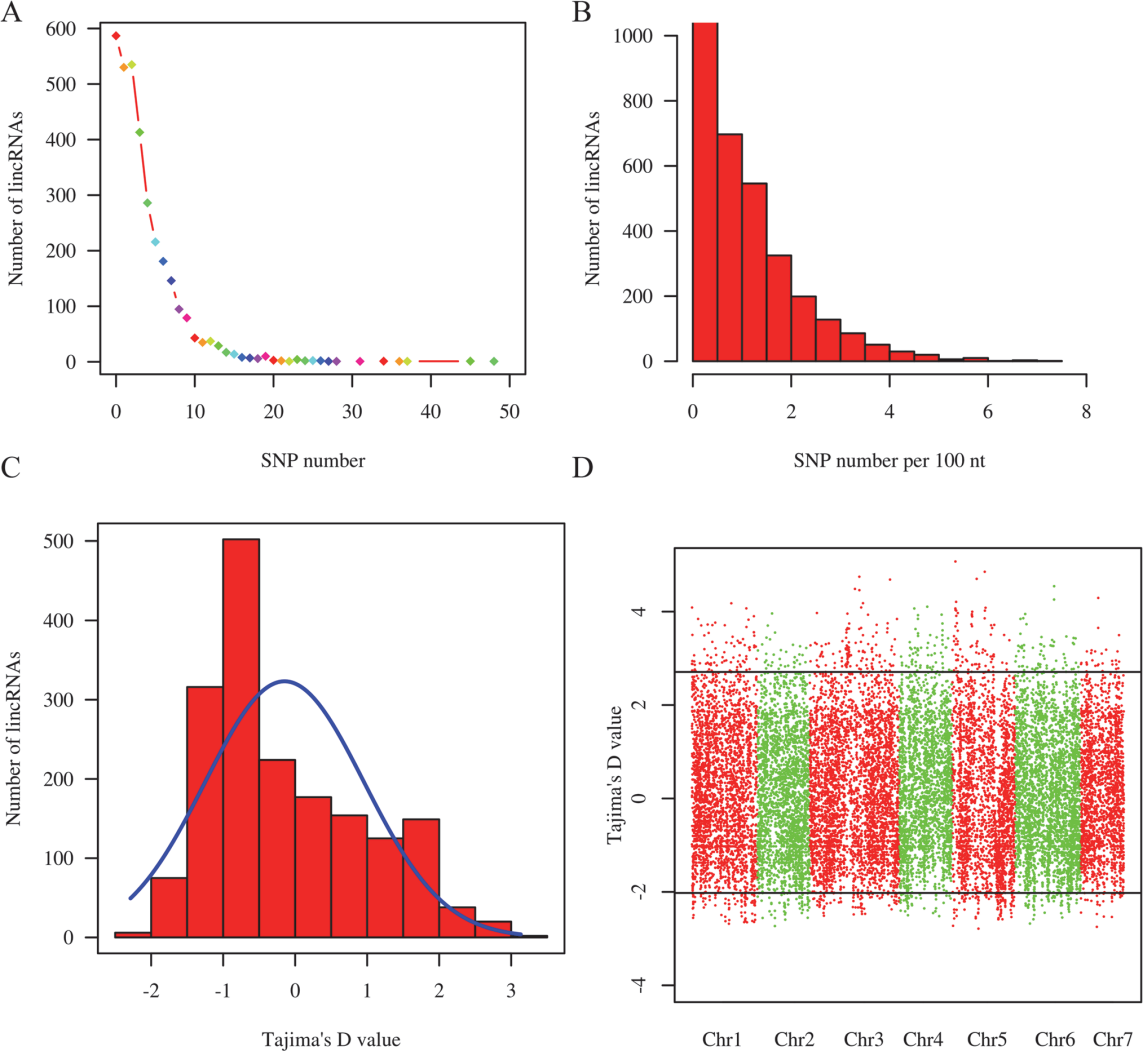
Distribution of SNPs and Tajima’s D values. (A) The distribution of SNPs for all lincRNAs. (B) The distribution of SNPs per 100 nt for all lincRNAs. (C) The distribution of Tajima’s D values for all lincRNAs from 102 cultivated cucumber accessions. (D) The distribution of Tajima’s D values for all intergenic regions from 102 cultivated cucumber accessions. The horizontal line indicates the 95% confidence interval.

We employed 102 cultivated cucumber accessions to conduct analyses of lincRNA polymorphisms and evolution [32]. Of the 3,274 investigated lincRNAs located at 3,298 loci, 11,923 SNPs were found in lincRNAs, comprising 1.12% of lincRNA nucleotides, with an average pairwise nucleotide diversity (π) of 0.00079439 ± 0.001338246 (mean ± SE of the mean). We found that 77.8% of the lincRNAs exhibit no more than 5 SNPs, and 587 (17.8%) display no SNPs (Fig. 7A). Furthermore, 2,763 of the lincRNAs (83.8%) presented ≤ 2 SNPs per 100 nt (Fig. 7B). The results showed that most of the lincRNAs show low divergence among the 102 cultivated cucumber accessions.

To examine the neutrality of lincRNAs in cucumbers, a widely used neutrality test (Tajima’s D) was performed for each lincRNA. Under the neutral equilibrium model (NE), the mean Tajima’s D is expected to be zero. A significant negative value of Tajima’s D indicates an excess of rare sequence variants relative to NE expectations, and recent positive selection is thus inferred. In contrast to positive selection, a significant positive value indicates balancing selection.

Based on the distribution of Tajima’s D for all of the lincRNAs, we could clearly see that while most of the lincRNAs are under purifying selection, there are 81 lincRNAs that display a significant probability (*p* < 0.05) of non-neutral patterns of sequence variation (Table 2, Fig. 7C). These included 36 lincRNAs showing significantly negative Tajima’s D values and 45 lincRNAs with significantly positive Tajima’s D values. Compared with the confidence interval obtained through multilocus analysis [average D = −0.068, 95% confidence interval (−2.022691; 2.707987)] calculated from the same sample of accessions at 22,423 noncoding loci (all the intergenic regions of the cucumber genome) (Fig. 7D), 16 of the 81 lincRNAs were outside of the 95% confidence interval (Table 2). Further analysis indicated that 9 of the 16 lincRNAs exhibiting a negative Tajima’s D values were under positive selection, and 7 of the 16 lincRNAs with a positive Tajima’s D value were under balancing selection, suggesting that at least 16 of the lincRNAs with a significant Tajima’s D might be a result of selection.

**Table 2:** Cucumber lincRNAs under positive selection and balancing selection.

**Gene_ID**	**Chr** ^a^	**Length**	**S** ^b^	**Pi** ^c^	**Tajima’s D** ^d^	**95% confidence interval** ^e^	**Peak expression tissue** ^f^
CU1NC10	Chr1	239	9	0.001105	−2.2659441**	No	NA
CU1NC88	Chr1	278	4	0.0062904	2.5505173*	Yes	Ovary1
CU1NC163	Chr1	291	2	0.0034695	2.4648383*	Yes	Root
CU1NC200	Chr1	501	7	0.0050127	2.0830965*	Yes	Root
CU1NC212	Chr1	636	4	0.0026174	2.3492285*	Yes	Root
CU1NC213	Chr1	544	4	0.0030601	2.3492285*	Yes	Root
CU1NC281	Chr1	203	3	0.0070937	2.7242963*	No	Ovary3
CU1NC336	Chr1	513	3	0.0029223	2.8775269*	No	Leaf
CU1NC346	Chr1	253	2	0.0039875	2.503925*	Yes	Root
CU1NC413	Chr1	294	2	0.0032004	2.2522393*	Yes	Root
CU1NC476	Chr1	256	3	0.0057236	2.7862235*	No	Ovary3
CU1NC531	Chr1	569	7	0.0003921	−1.9389757*	Yes	Ovary3
CU1NC532	Chr1	612	5	0.0001901	−2.0106222*	Yes	Ovary3
CU2NC602	Chr2	419	3	0.0005007	−1.9692466*	Yes	Ovary3
CU2NC712	Chr2	313	10	0.0039749	−2.0240647*	No	Ovary3
CU2NC844	Chr2	492	4	0.000271	−1.8212527*	Yes	Tendril
CU2NC897	Chr2	303	4	0.0012324	−1.8243111*	Yes	Ovary3
CU2NC903	Chr2	365	3	0.0040301	2.8203487*	No	Root
CU2NC943	Chr2	1005	7	0.000392	−1.9146939*	Yes	Ovary3
CU3NC998	Chr3	206	4	0.007585	2.0897819*	Yes	Ovary2
CU3NC1042	Chr3	261	2	0.0037901	2.4445273*	Yes	Root
CU3NC1046	Chr3	211	2	0.0047828	2.5080474*	Yes	Leaf
CU3NC1070	Chr3	224	2	0.0042939	2.3222843*	Yes	Ovary3
CU3NC1211	Chr3	342	5	0.0004525	−2.1717458**	No	Root
CU3NC1228	Chr3	205	4	0.0020915	−1.8572054*	Yes	Ovary3
CU3NC1230	Chr3	232	5	0.0004226	−1.9060085*	Yes	Root
CU3NC1394	Chr3	325	4	0.00515	2.3797784*	Yes	Ovary3
CU3NC1397	Chr3	271	3	0.0050691	2.5022962*	Yes	Ovary3
CU3NC1466	Chr3	359	2	0.0025722	2.1949941*	Yes	Male_flower
CU3NC1523	Chr3	271	6	0.0115769	2.5886134*	Yes	Root
CU4NC1592	Chr4	519	3	0.0028003	2.7559386*	No	Root
CU4NC1593	Chr4	270	5	0.0072616	2.2587333*	Yes	Root
CU4NC1605	Chr4	367	4	0.0045567	2.3688663*	Yes	Ovary3
CU4NC1606	Chr4	568	3	0.0021774	2.0839754*	Yes	Male_flower
CU4NC1644	Chr4	350	3	0.0037895	2.3348271*	Yes	Ovary3
CU4NC1659	Chr4	488	2	0.0019324	2.2553741*	Yes	Female_flower
CU4NC1667	Chr4	246	2	0.0037646	2.1717333*	Yes	Ovary1
CU4NC1767	Chr4	260	3	0.0058175	2.9329276*	No	Ovary2
CU4NC1781	Chr4	283	3	0.0044405	2.135983*	Yes	Ovary1
CU4NC1796	Chr4	196	5	0.0030814	−1.8400142*	Yes	Ovary3
CU4NC1800	Chr4	292	2	0.0033105	2.3304458*	Yes	Ovary3
CU4NC1802	Chr4	417	2	0.0023499	2.3860244*	Yes	Male_flower
CU4NC1868	Chr4	304	3	0.0041277	2.1525254*	Yes	Root
CU4NC1885	Chr4	516	3	0.0002247	−2.0113611*	Yes	Ovary2
CU5NC1977	Chr5	302	2	0.0033386	2.5229679*	Yes	Ovary3
CU5NC2008	Chr5	467	2	0.0019797	2.1919823*	Yes	Female_flower
CU5NC2111	Chr5	277	3	0.0008379	−1.9128665*	Yes	Ovary3
CU5NC2118	Chr5	385	2	0.0023619	2.1313885*	Yes	Stem
CU5NC2119	Chr5	605	2	0.001503	2.1313885*	Yes	Stem
CU5NC2199	Chr5	409	5	0.0002844	−2.0106222*	Yes	Tendril_base
CU5NC2215	Chr5	280	6	0.0006261	−1.8997299*	Yes	Male_flower
CU5NC2264	Chr5	361	2	0.002709	2.389199*	Yes	Ovary2
CU5NC2297	Chr5	750	3	0.0001277	−1.9040852*	Yes	Ovary2
CU5NC2308	Chr5	306	5	0.0004429	−2.0981125*	No	Ovary1
CU5NC2322	Chr5	289	5	0.0004683	−1.9736752*	Yes	Ovary1
CU5NC2333	Chr5	1185	12	0.0003154	−2.2046959**	No	Ovary3
CU5NC2398	Chr5	462	5	0.0006118	−1.839069*	Yes	Ovary2
CU5NC2405	Chr5	346	2	0.002886	2.4711995*	Yes	Tendril
CU5NC2413	Chr5	302	5	0.0003246	−1.9060085*	Yes	Leaf
CU5NC2414	Chr5	258	5	0.0004501	−1.8626232*	Yes	Tendril
CU6NC2488	Chr6	233	3	0.0013168	−1.7863936*	Yes	Female_flower
CU6NC2519	Chr6	435	5	0.0058552	2.1541265*	Yes	Female_flower
CU6NC2591	Chr6	368	8	0.0006311	−2.3781938**	No	Ovary1
CU6NC2592	Chr6	388	7	0.0003503	−2.0974517*	No	Ovary1
CU6NC2614	Chr6	376	3	0.0039007	2.7872349*	No	Root
CU6NC2664	Chr6	1414	5	0.0003119	−1.8432369*	Yes	Ovary3
CU6NC2673	Chr6	258	3	0.000859	−1.9415208*	Yes	Ovary1
CU6NC2686	Chr6	205	4	0.0080763	2.3471704*	Yes	Female_flower
CU6NC2748	Chr6	260	2	0.0035033	2.1110979*	Yes	Female_flower
CU6NC2798	Chr6	517	2	0.0002231	−2.0124695*	Yes	Ovary3
CU6NC2830	Chr6	1978	12	0.0001921	−2.273675**	No	Ovary3
CU6NC2833	Chr6	754	7	0.0036891	2.5633424*	Yes	Ovary3
CU6NC2881	Chr6	231	2	0.0043548	2.5263375*	Yes	Male_flower
CU7NC3029	Chr7	370	2	0.0026393	2.3991555*	Yes	Root
CU7NC3079	Chr7	453	6	0.0003417	−2.1717458**	No	Root
CU7NC3086	Chr7	202	2	0.0009368	−1.8691152*	Yes	Female_flower
CU7NC3093	Chr7	339	6	0.0018118	−1.8311426*	Yes	Root
CU7NC3094	Chr7	330	5	0.0014267	−1.9062613*	Yes	Ovary2
CU7NC3201	Chr7	345	3	0.0036221	2.130209*	Yes	Tendril
CU7NC3207	Chr7	465	3	0.0002485	−1.8638834*	Yes	Male_flower
CU7NC3276	Chr7	389	4	0.000346	−1.9751531*	Yes	Ovary3

* indicates a significance level of p<0.05

** indicates a significance level of p<0.01.

^a^ Chr: Chromosome where the lincRNA is located.

^b^ S: Number of polymorphic (segregating) sites.

^c^ Pi: Nucleotide diversity.

^d^ Tajima’s D calculated for lincRNAs from 102 cucumber accessions.

^e^ The threshold 95% confidence interval calculated for all cucumber intergenic regions from 102 cucumber accessions. "Yes" indicates that the Tajima’s D value for each lincRNA is within the 95% confidence interval, while "No" indicates that the Tajima’s D value for each lincRNA is outside of the 95% confidence interval.

^f^ Peak expression tissue: tissue with the highest expression level of lincRNAs. Ovary1: unexpanded ovary. Ovary2: expanded ovary (fertilized). Ovary3: expanded ovary (unfertilized).

## Discussion

High-throughput sequencing technologies (RNA-seq) allow the detection of novel types of transcripts, which often exhibit low expression levels. The use of a comprehensive set of transcripts constructed through the integration of multiple sources of data generated with different technologies enables the identification of types of RNA such as lincRNAs. In the present study, 3,274 lincRNAs from the cucumber genome were identified from different types of data, including EST, hi-seq and 454-seq data.

The number of lincRNAs found in cucumber is lower than the approximately 6,000 lincRNAs found in *Arabidopsis thaliana*, although these species display comparable genome sizes. The possible reason may be that we used stricter criteria to identify bona fide lincRNAs. First, several databases, including the nr, nt, and Swiss-Prot databases, were integrated into a pipeline with methods including BLAST and CPC to eliminate potential coding sequences in the putative lincRNA sets. Second, when subjected to BLAST searches against the nt database, lincRNAs that were significantly matched with sequences from chloroplasts and mitochondria were discarded. However, because evidence suggests that lncRNAs may come from the mitochondria in humans [33], the question of whether lincRNA genes exist in the mitochondria or chloroplasts of plants needs to be further explored in the future.

To investigate the function of cucumber lincRNAs, a lincRNA-mRNA co-expression network was constructed and employed to predict the function of cucumber lincRNAs using hub-based and module-based methods. In total, the function of 165 lincRNAs, including 96 lincRNAs that overlapped in the two methods, could be inferred. The reason for the low rates of the prediction of lincRNA function may be that lincRNAs participate in many biology processes through different mechanisms and function in different ways. Therefore, the majority of lincRNAs are not necessarily co-expressed with mRNAs, and better strategies should be developed to mine lincRNA function.

The regulatory mechanism of cucumber lincRNAs is unknown. Based on the relationship between miRNAs and lincRNAs, we predict that 10 lincRNAs are potential targets or target mimics of 17 miRNAs with high expression scores. Our results provide insight into lincRNA regulation mechanisms and will hopefully be validated in the future.

Compared with mRNAs, plant lincRNAs are usually less conserved and evolve more rapidly. Genome-wide lincRNA evolution in plants has not been reported. Based on the results of Tajima’s D test in 102 cucumber accessions, we infer that most lincRNAs are under purifying selection, with 16 lincRNAs under positive or balancing selection.

In summary, our results provide a rich source of information for research into the function of lincRNAs in cucumber. We provide many insights into cucumber lincRNAs, but more work is necessary to understand the function and evolution of cucumber lincRNAs.

## Materials and Methods

### Cucumber genomic and transcriptome data, including EST, 454-seq, and Hi-seq data

The cucumber genome and annotation file (version2) were downloaded from the Cucurbit Genomics Database (ftp://www.icugi.org/pub/genome/cucumber/Chinese_long/v2/cucumber_ChineseLong_v2_genome.fa.gz and ftp://www.icugi.org/ pub/genome/cucumber/Chinese_long/v2/cucumber_ChineseLong_v2.gff3.gz) [34]. EST sequences (version3.0) were also downloaded from the above website.

The transcriptomes of young cucumber fruits at five ages sequenced through 454-pyrosequencing (454-seq) were downloaded from the NCBI database under GEO accession number GSE39310 [35].

Illumina high-throughput sequences (Hi-seq) were downloaded from the NCBI database under accession number SRA046916 [36]. These data are paired-end reads with read lengths of 75 bp and come from ten cucumber tissues: root, stem, leaf, male flowers, female flowers, ovary, expanded fertilized ovary (7 days after flowering), expanded unfertilized ovary (7 days after flowering), base of the tendril, and tendril. *Do novo* assembly was performed using Trinity [37].

### Pipeline for lincRNA identification

The pipeline employed for lincRNA identification was as follows: (1) Assembled transcripts were aligned with the cucumber genome and were retained when all nucleotides were mapped to the genome without mismatches using BLAT (min Identify = 100) [38]. (2) Transcript units (TUs) that overlapped with annotated genes or NATs (natural antisense transcripts) were discarded from further analysis. The remaining TUs were considered intergenic TUs. (3) TUs that overlapped repeat elements identified by RepeatMasker were also discarded. (4) TUs located within the 500 bp flanking regions of annotated protein-coding genes were removed. (5) TUs were scanned with Ugene (http://ugene.unipro.ru/) using the “find-orfs” function to find open reading frames (ORFs) with the following parameters: require-init-codon = false, min-length = 300. TUs containing ORFs with a length of 300 nt or more were eliminated. (6) TUs with lengths of less than 200 nt were discarded. (7) The Swiss-Prot database was used to filter out TUs with matched protein sequences (BLASTX, E-value ≤ 1e-10). (8) The CPC (coding potential calculator) was employed to identify TUs with coding potential [28]. (9) The nr (non-redundant protein sequence) database of NCBI was used to discard TUs with homologous sequences with a cutoff E-value of ≤ 1e-10 employing BLASTX. (10) The nt (nucleotide sequence) database of NCBI was used to discard lincRNAs containing CDS employing BLASTN (E-value ≤ 1e-10). (11) The Rfam database was used to discard housekeeping RNAs, such as tRNAs, rRNAs, snRNAs, and snoRNAs (E-value ≤ 1e-10) with BLASTN. (12) The CD-HIT tool was used to cluster lincRNAs with an identity of 95%, and the longest sequence in the cluster was selected for further analysis [39].

### Conservation and expression analysis of lincRNAs

The lincRNA homologs in 10 representative plant genomes were investigated based on sequence similarity. The genomes of *P*. *patens*, *S*. *moellendorffii*, *P*. *abies*, *O*. *sativa*, *Z*. *mays*, *A*. *thaliana*, *P*. *trichocarpa*, and *V*. *vinifera* were downloaded from phytozomes (v9.1) (http://www.phytozome.net/). The watermelon and melon genomes were obtained from the Cucurbit Genomics Database (http://www.icugi.org/) and the melon genome database (http://melonomics.net/), respectively. The lincRNA sequences were aligned against these 10 plant genomes with BLASTN [40]. The cutoff threshold for significant hits was an E-value of < 1e-10 and coverage of > 20% of matched regions.

Hi-seq data from 10 cucumber tissues were used for expression analysis of lincRNAs. Reads were mapped to lincRNAs with Bowtie [41], and the expression levels (Reads per Kilobase per Million Reads, RPKM) of lincRNAs were quantified using a Perl script. The lincRNAs were classified into four levels: low (exp_*max*_ ≤ 5 RPKM), moderate (exp_*max*_ ≥ 5 RPKM and exp_*max*_ < 10 RPKM), high (exp_*max*_ ≥ 10 RPKM and exp_*max*_ < 20 RPKM), and very high (exp_*max*_ ≥ 20 RPKM).

### Tissue-specific expression

The tissue specificity of the observed expression patterns was evaluated according to the tissue-specific index, which ranges from 0 for house-keeping genes to 1 for tissue-restricted genes [30]. The index was calculated using the following formula:$\text{tissue-specific index =}\frac{\sum_{i=1}^{n}\left(1-\frac{\exp_{i}}{\exp_{\max}}\right)}{\text{n}-1}$, where *n* is the number of tissues; exp_*i*_ is the expression value of each lincRNA in tissue, *i*; and exp_*max*_ is the maximum expression value of each lincRNA among all tissues. The expression value of each lincRNA is counted as the RPKM (reads per kilobase per million reads). The lincRNAs showing a tissue-specific index > 0.9 were considered to display tissue-specific expression. The lincRNA expression data were clustered and displayed using heatmap.2 in R packages.

### miRNA target prediction in cucumber lincRNAs

To search the lincRNAs for potential miRNA targets, 3,274 lincRNAs and 64 cucumber miRNAs [42] were uploaded into psRobot, a widely used online miRNA target prediction tool, with moderate parameters (penalty score threshold = 2.5, five prime boundary of essential sequence = 2, three prime boundary of essential sequence = 17, maximal number of permitted gaps = 1, position after which with gaps permitted = 17) (http://omicslab.genetics.ac.cn/psRobot/) [43].

### Construction of the lincRNA-mRNA co-expression network

The Hi-seq data collected from ten tissues were used to construct a lincRNA-mRNA co-expression network [36]. The construction method was similar to that of Liao [44]. In general, the pipeline for constructing the co-expression network was as follows: (1) The genes, including mRNAs and lincRNAs, whose variances ranked in the top 75% of expression profiles were retained. (2) The *p*-values of Pearson correlation coefficient (*Pcc*) was calculated for each pair of genes using Fisher’s asymptotic test in the *WGCNA* library of R [45], and were adjusted using the Bonferroni correction method. (3) Co-expression relationships showing adjusted *p*-values of less than 0.05 and ranking in the top 5% and bottom 5% of *Pcc* were selected for further analysis. The Bonferroni multiples test was executed using the *multtest* package of R (*multtest*: Resampling based multiple hypothesis testing, 2014. R package version: 2.20.0). Cytoscape was employed for visualization of the co-expression network [46].

### Prediction of cucumber lincRNA function

LincRNAs were annotated using hub-based and module-based methods that have been widely applied [44]. The hub-based method is used to annotate a gene based on the enrichment of its immediate neighborhood. In the lincRNA-mRNA co-expression network, a lincRNA with at least 10 mRNA partners can be regarded as one hub-gene. Each hub-gene and its mRNA partners constitute one lincRNA subnet. The function of hub-genes (lincRNAs) can be predicted based on the GO (Gene Ontology) enrichment of mRNAs within all lincRNA subnets. Online tools in the cucurbit genomics database (http://icugi.org/cgi-bin/ICuGI/tool/GO_enrich.cgi) were used to perform the GO enrichment analysis. The FDR-corrected cutoff *p*-value for significantly represented GO terms was set as 0.05. Under the module-based method, the MCL algorithm was used to search for modules in the co-expression network [47]. For each module, a method similar to the hub-based method was used to predict the functions of lincRNAs within modules.

### Evolutionary analysis of lincRNAs

The SNP files of 102 cucumber accessions can be downloaded from the Cucumber genome database (ftp://www.icugi.org/pub/reseq/cucumber/SNP/) [32]. All lincRNAs and intergenic regions from different cucumber accessions were extracted based on the reference genome and SNP files for each cucumber accession using in-house-generated Perl scripts. The numbers of SNPs for each gene were calculated by counting the sum of sites with different bases (excluding gaps or N). Variscan was used to carry out the Tajima’s D analysis (options: useMuts = 1, runmode = 12, completeDeletion = 0, fixnum = 0, numNuc = 4) [48]. The *p*-value of Tajima’s D was calculated using Dnasp with the default parameters [49].

## Supporting Information

S1 Fig LincRNA distribution among seven chromosomes. The black bars on every chromosome show the positions of lincRNAs.
(TIF)

S2 Fig Number of conserved lincRNAs in 8 representative plants. (TIF)

S3 Fig Density graphs for comparison of expression between lincRNAs and mRNAs in different tissues. Red lines represent lincRNAs; green lines represent mRNAs.
(TIF)

S4 Fig Prediction of the function of overlapping lincRNAs through hub-based and module-based methods. (A) The main molecular functions (MF) and (B) cellular components (CC). The X-axis indicates the numbers of enriched mRNAs.
(TIF)

S1 Table Transcriptome data used to predict lincRNAs. (XLS)

S2 Table Genomic information for the identified lincRNAs (GFF format). (XLS)

S3 Table Conservation of lincRNAs across three genomes, including cucumber, melon and watermelon. (XLS)

S4 Table Tissue-specific expression of lincRNAs. (XLS)

S5 Table The functions of overlapping lincRNAs predicted through the hub-based method. (XLS)

S6 Table The functions of overlapping lincRNAs predicted through the module-based method. (XLS)
