# Supplementary figures and images for: Genome-Wide Identification, Characterization and Evolutionary Analysis of Long Intergenic Noncoding RNAs in Cucumber
Source: PLoS One. 2015 Mar 23;10(3):e0121800. doi: 10.1371/journal.pone.0121800 (PMC4370693; doi:10.1371/journal.pone.0121800)

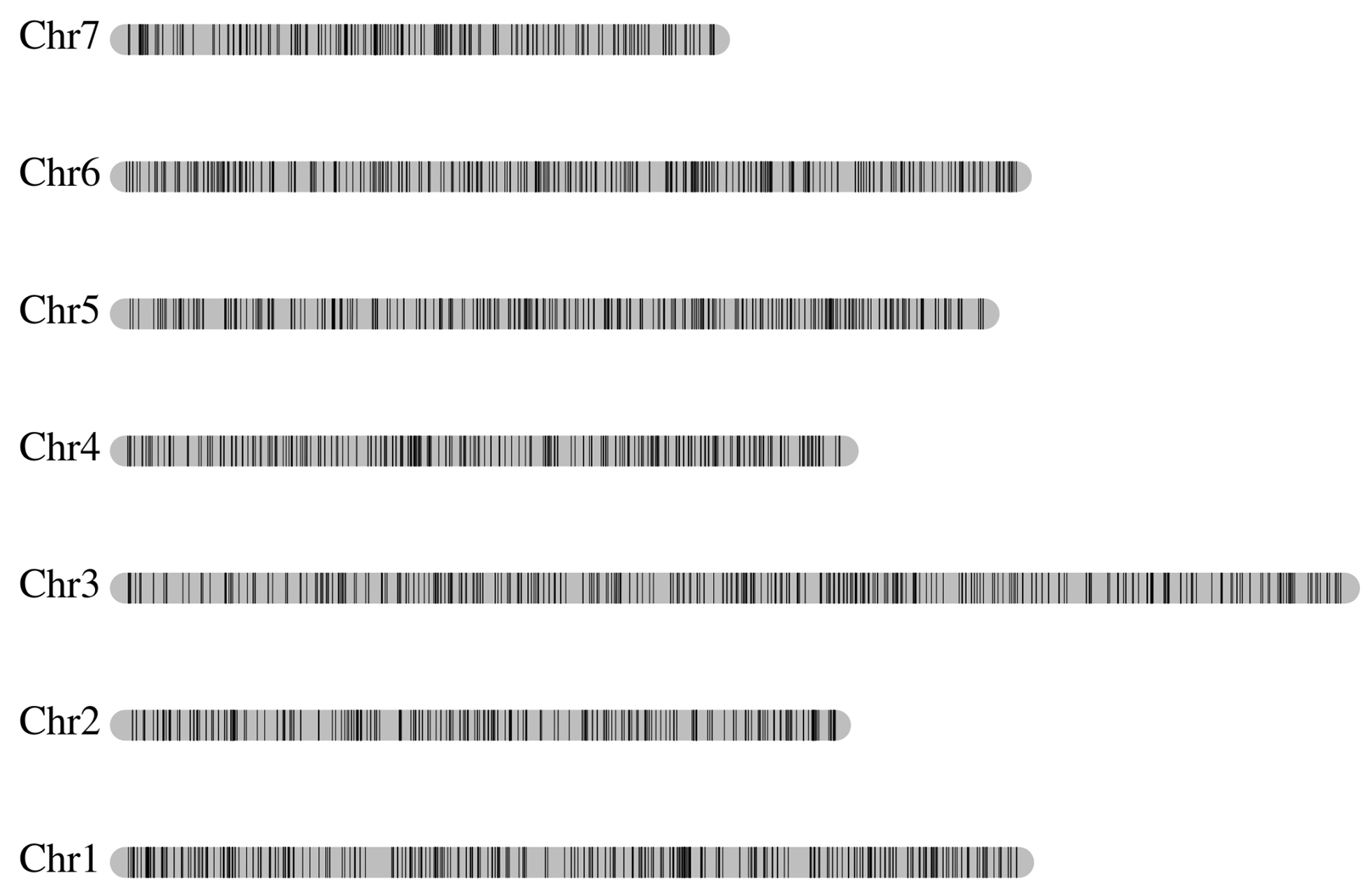

Supplement: S1 Fig — The black bars on every chromosome show the positions of lincRNAs. (TIF) [file pone.0121800.s001.tif]

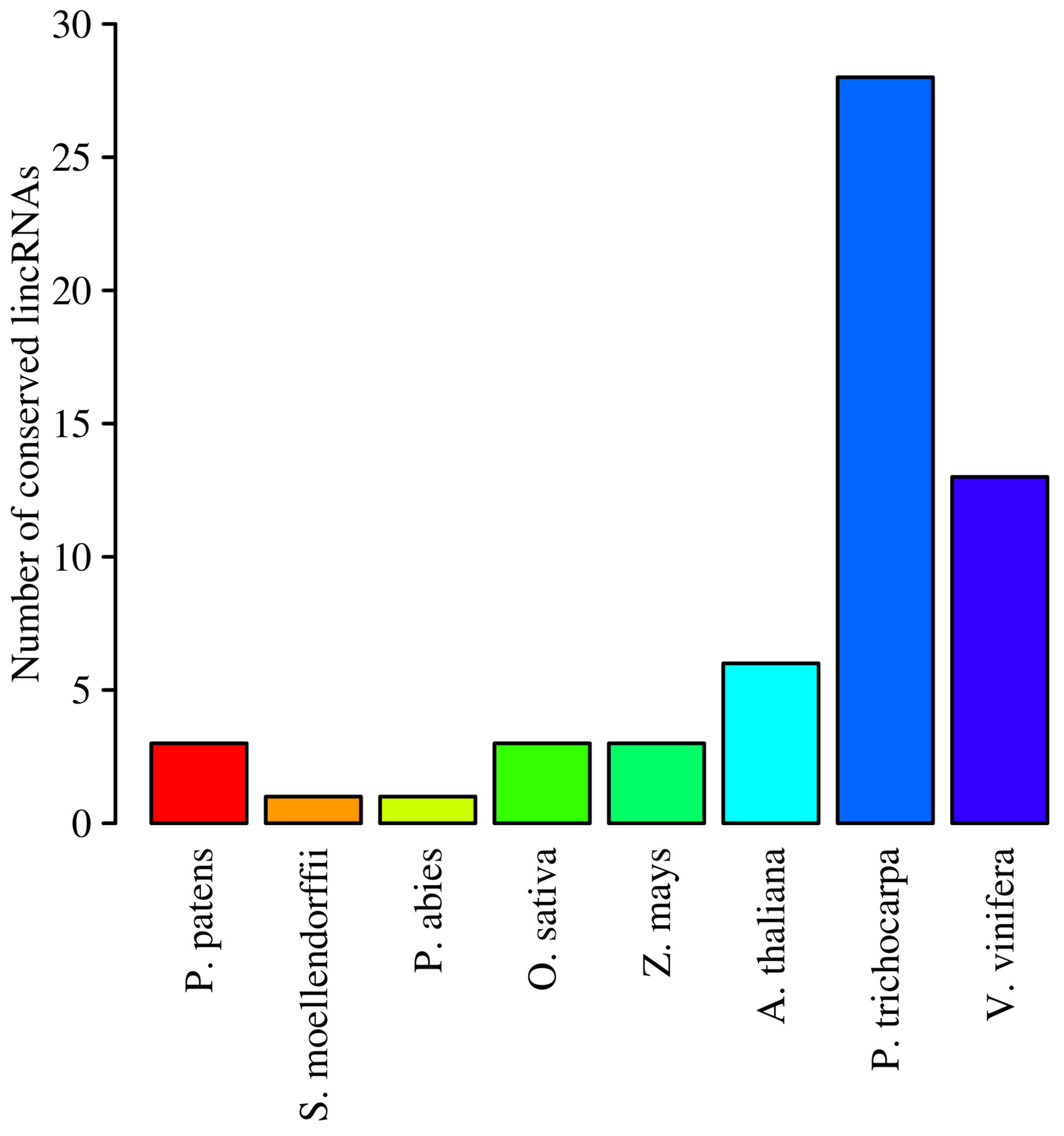

Supplement: S2 Fig — (TIF) [file pone.0121800.s002.tif]

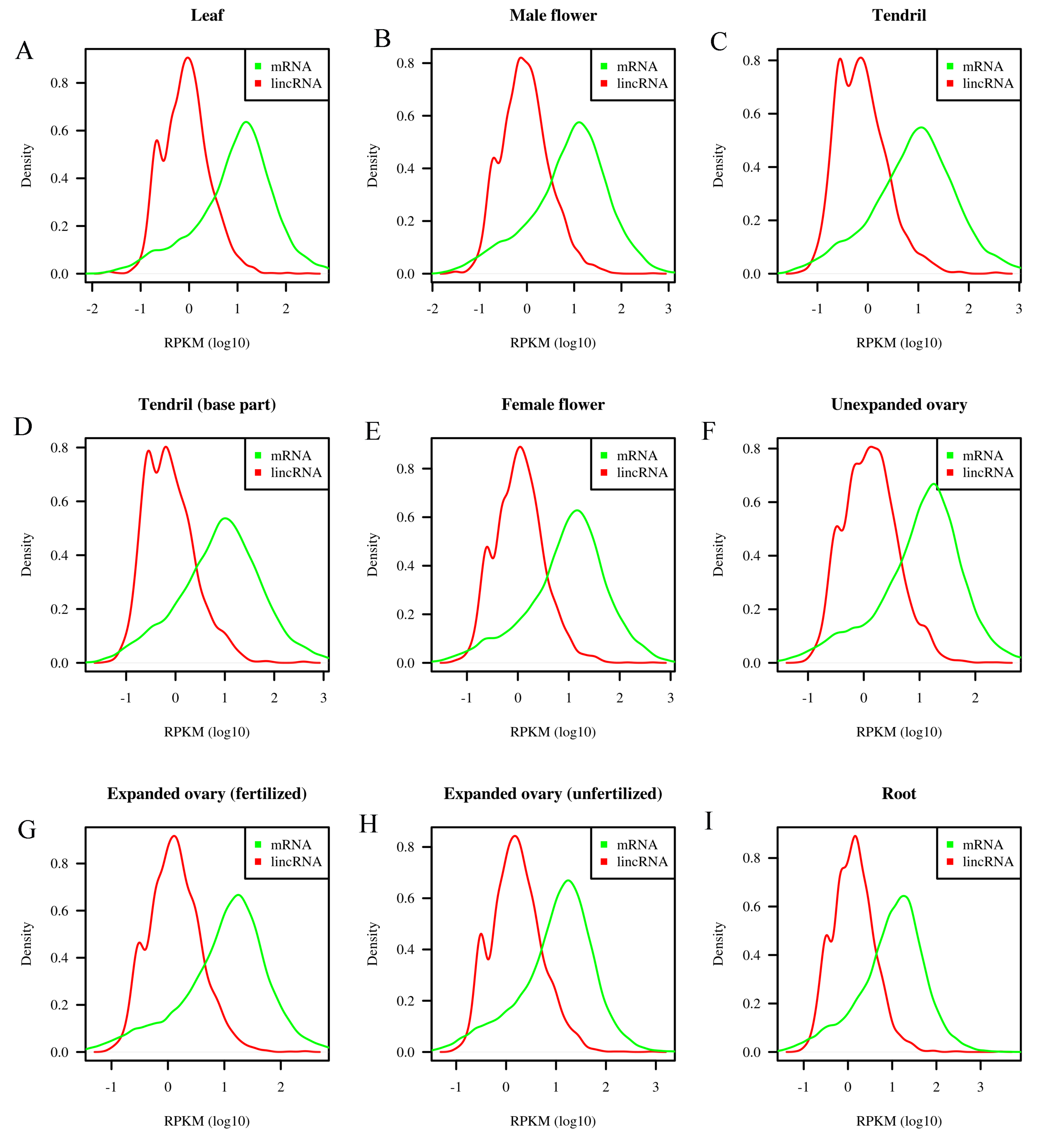

Supplement: S3 Fig — Red lines represent lincRNAs; green lines represent mRNAs. (TIF) [file pone.0121800.s003.tif]

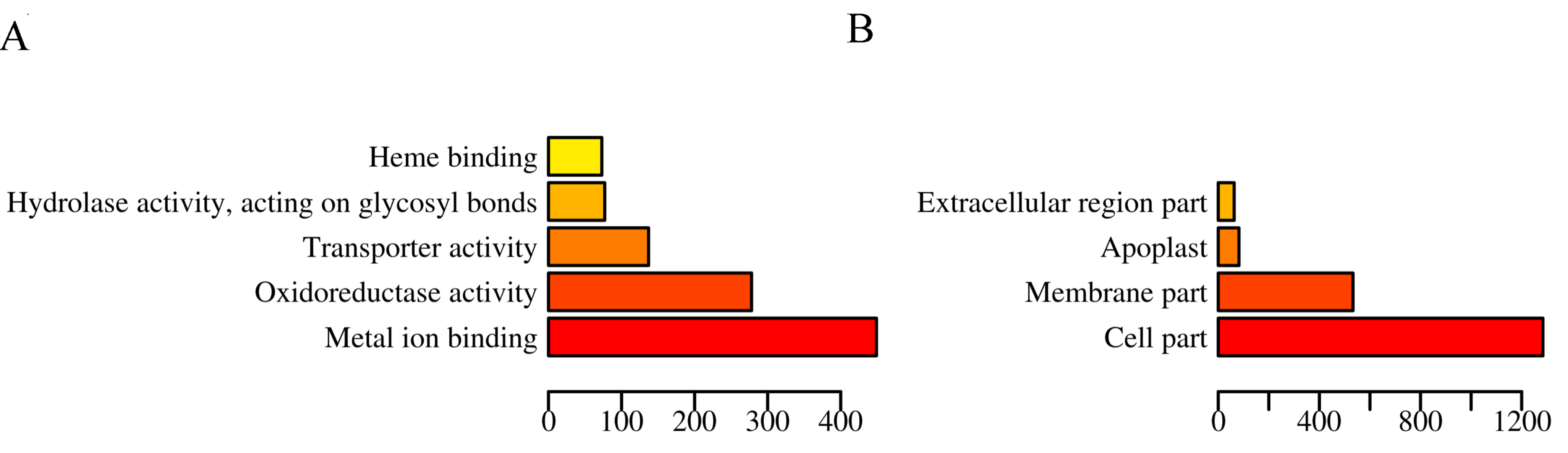

Supplement: S4 Fig — (A) The main molecular functions (MF) and (B) cellular components (CC). The X-axis indicates the numbers of enriched mRNAs. (TIF) [file pone.0121800.s004.tif]
